# Microfluidic investigation of the effect of graphene oxide on mechanical properties of cell and actin cytoskeleton networks: experimental and theoretical approaches

**DOI:** 10.1038/s41598-021-95624-0

**Published:** 2021-08-10

**Authors:** Mohammad Ghorbani, Hossein Soleymani, Hadi Hashemzadeh, Saeed Mortezazadeh, Mosslim Sedghi, Seyedehsamaneh Shojaeilangari, Abdollah Allahverdi, Hossein Naderi-Manesh

**Affiliations:** 1 Department of Biophysics, Faculty of Biological Science, Tarbiat Modares University, 14115-154 Tehran, Iran; 2 Department of Nanobiotechnology, Faculty of Biological Science, Tarbiat Modares University, 14115-154 Tehran, Iran; 3 Biomedical Engineering Group, Department of Electrical Engineering and Information Technology, Iranian Research Organization for Science and Technology (IROST), P.O. Box 33535111, Tehran, Iran

**Keywords:** Biophysics, Cancer, Cell biology

## Abstract

Biomechanical and morphological analysis of the cells is a novel approach for monitoring the environmental features, drugs, and toxic compounds’ effects on cells. Graphene oxide (GO) has a broad range of medical applications such as tissue engineering and drug delivery. However, the effects of GO nanosheets on biological systems have not been completely understood. In this study, we focused on the biophysical characteristics of cells and their changes resulting from the effect of GO nanosheets. The biophysical properties of the cell population were characterized as follows: cell stiffness was calculated by atomic force microscopy, cell motility and invasive properties were characterized in the microfluidic chip in which the cells are able to visualize cell migration at a single-cell level. Intracellular actin was stained to establish a quantitative picture of the intracellular cytoskeleton. In addition, to understand the molecular interaction of GO nanosheets and actin filaments, coarse-grained (CG) molecular dynamics (MD) simulations were carried out. Our results showed that GO nanosheets can reduce cell stiffness in MCF7 cells and MDA-MB-231 cell lines and highly inhibited cell migration (39.2%) in MCF-7 and (38.6%) in MDA-MB-231 cell lines through the GO nanosheets-mediated disruption of the intracellular cytoskeleton. In the presence of GO nanosheets, the cell migration of both cell lines, as well as the cell stiffness, significantly decreased. Moreover, after GO nanosheets treatment, the cell actin network dramatically changed. The experimental and theoretical approaches established a quantitative picture of changes in these networks. Our results showed the reduction of the order parameter in actin filaments was 23% in the MCF7 cell line and 20.4% in the MDA-MB-231 cell line. The theoretical studies also showed that the GO nanosheet–actin filaments have stable interaction during MD simulation. Moreover, the 2D free energy plot indicated the GO nanosheet can induce conformational changes in actin filaments. Our findings showed that the GO nanosheets can increase the distance of actin-actin subunits from 3.22 to 3.5 nm and in addition disrupt native contacts between two subunits which lead to separate actin subunits from each other in actin filaments. In this study, the biomechanical characteristics were used to explain the effect of GO nanosheets on cells which presents a novel view of how GO nanosheets can affect the biological properties of cells without cell death. These findings have the potential to be applied in different biomedical applications.

## Introduction

The mechanical properties of the living cell as a biological parameter are a novel vision in cell biology. In many diseases, the alteration of the mechanical properties of cells is an important parameter during disease progression^1–8^. The stiffness of biological cells is a crucial parameter in many fundamental cellular processes such as morphogenesis, differentiation, and migration^4,9,10^. This property is correlated to the cytoskeleton, especially actin filament and cellular bio-membrane. The previous results indicated that the actin bundle has the main role in the stiffness of healthy cells whereas the stiffness of malignant cells significantly decreases due to lack of actin bundle during cancer progression^1,7,11,12^.

Cell stiffness which is related to the actin bundle shows that GO interaction with cellular components is an important factor in the cytotoxicity mechanism of GO^13^. The previous theoretical and experimental reports have shown that GO has toxicity effects on orientation and intensity of actin which differs between healthy and malignant cells^13^.

In recent years, the commercial use of nanomaterials has been significantly increased especially in medical applications^14,15^. Carbon nanomaterials with unique structural and physical properties have gained a great deal of attention in a large range of biomedical fields such as cellular imaging and drug delivery^16,17^.

The interaction of GO with biological macromolecules is an important issue in biological and medical applications due to diverse applications in biomedical researches^17–19^. However, the biological effects of GO and interaction with biological material are still unclear.

Recent studies show that the GO nanosheet affects bacterial and mammalian cells via disrupting cell membranes and the actin filament of cells. The GO induces physical damages in the bio-membrane by binding to the surface of the cell membrane^20^. The GO sheet could create a pore in the membrane in which its toxicity is related to these pores that are created on the cell membrane^20,21^. In addition, to penetrate the cell membrane, the GO sheet can bind to actin filament and disrupt protein–protein interaction, and separate protein dimer^13^.

Although, the effect of GO on the biological properties of the cells and the molecular mechanism of biological effects of GO has been widely studied, however, the biophysical view of the GO effect is open for further investigation. Mechanical properties of the cells especially the stiffness change is one of the symptoms of cancer progression^22,23^. Therefore, cell stiffness could be an appropriate candidate for the diagnostic and therapeutic purposes of diseases such as diabetes and cancers.

In this study, we have investigated the effect of GO on the biophysical properties of cancer cells such as cell stiffness, cell morphology, and actin orientation. In order to investigate this effect, physical parameters such as cell stiffness, organization of actin bundle, migration properties of cells and EM transition were calculated. Atomic Force Microscopy (AFM) was carried out to calculate the effect of GO on Young’s Modulus in cancer cells. The organization of the actin bundle in living cells was observed with a fluorescence inverted microscope. In addition, we designed a microfluidic device to investigate the migration properties and epithelial-mesenchymal (EM) transition at the single-cell level.

Traditional techniques show a major deficiency in single-cell migration, migration velocity, and cell heterogeneity^16,24^. The favorable features for cell migration devices include migration velocity at single-cell, cell heterogeneity, a small number of sample cells, and quantitative migration data. The platform presented here is able to visualize quantitative migration data and heterogeneity of cells at the single-cell level. We have designed and fabricated a microfluidic device to observe the relationship between stiffness changes and migration and EM transition properties of cells at the single-cell level (Fig. S1).

## Materials and methods

### Photolithography

The microfluidic architecture was designed using AutoCAD software and fabricated through photolithography followed by molding processes. Initially, to make the master for 10 μm channels, spin-coated SU-8 2050 photoresist on silicon has been used and baked at 95 °C for 2 min, the silicon and photo-mask were aligned and exposed to UV for 45 s. The silicon was developed using the developer solution for 3–5 min. After that, the silicon wafer was cleaned using DI water and dried with nitrogen gas. The silicon master was baked at 150 °C for 3 min. The master for 40 μm main channels was prepared by spin-coated SU-8 on a silicon wafer and exposed to UV for 45 s and developed by SU-8 developer and was hard-baked at 150 °C for 3 min.

### Preparation of microfluidic device

The migration device was formed from a single layer of PDMS (polydimethylsiloxane), and a glass slide. The two masks were used to fabricate the two heights, the main channels (40 µm) and migration channels (10 µm). The PDMS layer bound to glass by inducing the hydrophilic group using a plasma cleaner. Before cell loading, fibronectin solution (50 ng mL^−1^) was used to coat the PDMS surface in the migration chip to enhance cell adhesion. The right main channel was rinsed off to remove extra fibronectin by PBS after 1 h. Subsequently, cells were loaded into the cell chamber and incubated for 12 h in standard condition. The chemoattractant gradient was established and cells migrated during microchannels in the microfluidic device.

### Cell loading

The cells detached from the cell culture flask and the cells solution was centrifuged and re-suspended in free FBS-media at half a million cells mL^−1^. After that, 20 μL of the cells solution added to the main chamber from the inlet without applied external pressure. Cells gently entered the main chamber for cell seeding and attached to the surface after 12 h.

### Cell viability analysis on-a-chip

We used the Calcein AM/ PI Kit to observe cell viability in our microfluidic chip. In this test, the live and dead cells were stained as green and red, respectively^25^. After cell loading, cell adhesion to the substrate in the microfluidic device, and subsequently achieving appropriate morphology, the chip was washed using PBS solution. Then the Calcein AM / PI (Thermo fisher scientific) was added into the main chamber for 20–30 min at 37 °C. Cell viability has been calculated as the percentage of green cells to all of the cells. For cell viability assay, four replicate tests were carried out for each cell line.

### Cell culture and sample preparation

The MDA-MB-231 and MCF-7 breast cell lines were obtained from Royan Institute, Tehran, Iran, and cultured in high glucose DMEM with 10% FBS (Gibco BRL Life Technologies Inc., USA) and 1% penicillin/streptomycin (Invitrogen). Both cell lines were cultured in an incubator at 37 °C and 5% CO_2_.

### Preparation and characterization of graphene oxide (GO)

The GO nanosheets were synthesized by Modified Hummer’s method which described in our previous study and the characterization of GO nanosheets shows in Figs. S2, S3, S4^26^. The functional groups of GO nanosheets were characterized by Fourier transform infrared (FTIR) spectroscopy (Bruker Tensor 27 Spectrometer Bruker, Germany) from 4000 to 400 cm − 1, at room temperature using dried GO samples. Raman spectra of GO samples were measured by Raman spectrometer (Teksan company, model: Takram P50C0R10, Iran) with an Nd:YAG laser excitation wavelength of 532 nm. The XRD spectra were recorded by Philips, X'Pert MPD 40 kV, 40 mA, Cu. The UV–Vis spectra were recorded by using a Perkin Elmer Lambda 25 UV–Visible. Raman spectra were recorded using a Raman system (TakRam N1-541-Teksan) with excitation from a laser beam (532 nm) at a low power level (5 mW). The images were taken using an Olympus IX81 fluorescence microscope (Olympus, Hicksville, NY, USA).

The morphology and surface of GO samples were characterized by AFM at contact mode. The GO nanoparticles were dissolved in PBS to prepare 100 µg mL^−1^, of the GO solution and then ultrasonically processed. The droplet of GO solution was put on the glass and dried at room temperature. The surface images of the GO sheet were taken by Multi-Mode AFM (ARA Research, Iran) in tapping mode. Tapping mode is used to capture the height and phase images of 512 × 512 pixel size at a scan speed of 1.0 lines per second. The spring constant and diameter of the probe tip were 0.06–0.40 N/m and 16 nm, respectively. All the images are processed using Imager analysis software.

### Toxicity/viability assay

The cell viability of both cell lines in the presence of different concentrations of GO nanoparticles was measured by tetrazolium dye. MTT assay is a non-toxic, easy, and calorimetric indicator that was performed as previously described^27^. In the current study, 5000 cells were seeded in each well of a 96-well plate (SPL Life Sciences Co., Ltd. Korea). After 12 h, the cells were treated with the GO nanosheets in different concentrations (0, 1, 10, 50, 10, 200 µg mL^−1^) and placed in an incubator with the standard condition. After 24 h and 48 h, the medium was removed and added 100 μL of MTT solution (final concentration 0.5 mg mL^−1^) to each well for 4 h. The liquid was aspirated and the formazan precipitates were dissolved in DMSO/ethanol solution. The optical densities were measured at 570 nm using a microplate reader (BioTek Instruments, Inc., Winooski, VT, USA). For the MTT assay, six replicate tests were carried out for each concentration. MCF-7 and MDA-MB-231 cell lines without the GO nanosheets were considered as the control group.

### Calculation of Young’s modulus

To reach the ideal cell adhesion condition on the substrate, 10,000 cells were seeded on coverslips and incubated in medium culture (DMEM High Glc, 10% FBS, 1% penicillin/streptomycin) at 37 °C and 5% CO_2_ for 24 h. After that, old media was replaced with 1 mL fresh media containing three different concentrations of Graphene oxide: 0, 1, and 5 μg mL^−1^ were added to cells and incubated for another 24 h. Afterward, Young’s modulus of living cells was measured by AFM (CoreAFM, Nanosurf, Switzerland).

All of the data were obtained in liquid mode using the AFM (CoreAFM, Switzerland). The force-distance curve (F-D curves) was analyzed using the AtomicJ program. The old media was replaced by fresh media containing GO nanosheets. After that, the image of cells was taken by AFM device. In the following, for each cell, a window and a set of points were selected to measure the F-D curves. We used a very low loading rate of 1 Hz, and a rational indentation depth of 500 nm. The probe with a diameter of 20 nm (spring constant was 0.02 N/m) was used to measure the F-D curves (50 ~ 70) for each group. The F-D curve was fitted by the Hertz model:

$$ F = \frac{4}{3}\frac{E}{{1 - \gamma^{2} }} \sqrt {R\delta^{{\left( \frac{3}{2} \right)}} } $$

where F is the force, E is the Young’s modulus, γ is the passion ratio (we choose γ = 0.4 for all cases in this work), R is the radius of indenter and $\delta$ is the indentation depth. All of the data were processed by the AtomicJ program^28^. The indentation depth and loading rates were 500 nm and 1 Hz, respectively. The spring constant and diameter of the probe tip were 0.02 N/m and 20 nm, respectively^11^. The F-D curves were fitted using the Hertz model and all of the data were analyzed using the Origin 2016 program^29^.

### Actin staining using fluorescence dye

Cells were seeded on coverslips and cultures in presence of 0, 1, and 5 μg mL^−1^ of the GO nanosheets solutions for 24 h. After that, the coverslips were washed by PBS 2 to 3 times and fixed by using 4% formaldehyde in PBS at room temperature for 10 min. Cells were permeabilized by 0.1% Triton X-100 for 2–3 min and added 1% bovine serum albumin (BSA) to reduce non-specific binding. For fluorescence staining, the cells were incubated for 20 min with Phalloidin-FITC (1:40) and Hoechst (10 µg mL^−1^ in PBS) was used to stain actin filaments and nuclei and examined using the inverted fluorescence microscope Olympus IX81 equipped with DP72 camera. Actin was labeled with Alexa Fluor™ 488 Phalloidin (A12379, Invitrogen). FITC fluorescence was excited at 495 nm and measured at 519 nm. Hoechst fluorescence was excited at 392 nm and measured at 440 nm.

### Coarse-grained graphene oxide model

#### Coarse-grained simulation

The CG molecular dynamics simulations were carried out using Gromacs 2019 the Martini force field version 2.2. The CG structure of actin polymer was generated using the martinize.py program on the PDB structure obtained from RCSB (ID: 1M8Q). The CG structure of the GO nanosheet has been created by a 2:1 mapping of the non-hydrogen atoms of GO nanosheet. The force field parameters of GO were obtained based on previous works^30^ which developed a CG model of graphene nanosheet. For this aim, the CG beads of graphene (SG4) were randomly replaced by oxygen beads (SP1) which were CG models of epoxy and hydroxyl groups of GO. The CG models of GO involved 48% oxygen bead (SP1) and 52% CG bead of graphene (SG4). For building CG models, we used a home code written in Python, which was tested in our previous work to lipid modeling and is available from this link: https://github.com/saeedMRT/scg4py^31^.

The CG structures were inserted into a cubic box (25 nm × 25 nm × 25 nm ) with periodic boundary conditions in 3 dimensions and 5000 steps of energy minimization were performed using the steepest descent algorithm^32,33^. The system temperature was stabilized at 300 K using the V-rescale method with a time constant of 1 ps. The Parrinello-Rahman method was used for pressure coupling of the system at 1 bar with a time constant of 15 ps^32^. The fast smooth Particle-Mesh Ewald (PME) method was applied for calculating the Coulombic interactions with cutoff 1.2nm^34^. The force switching method with a cutoff of 1.2 nm was used for calculating the Lennard–Jones interactions. All systems were simulated for 1.5 µs with a time step of 30 fs where the coordinates of particles were extracted every 200 ps for analysis.

#### Simulation details

The MD simulations were performed using Gromacs 2019 and martini force fields^35,36^. The actin polymer was obtained from RCSB (ID: 1M8Q) and GO nano-sheets were built using our Python package. The force field was used for graphene oxide from previous studies^30,37^. The GO nanosheets were inserted into a cubic box ( 25 nm × 25 nm × 25 nm ). The systems were minimized using the steepest descent algorithm. The temperature of systems was equilibrated using Berendsen thermostat at 300 ºK and pressure equilibration was performed using Berenson barostat^32,33^. The periodic boundary conditions were used in all directions. The PME method was applied in long-range interactions and cutoff 1.2 nm was used for vdW (Van der Waals) interactions calculation. The time step of 30 fs was used, and coordinates were collected every 200 ps. The simulation time for all systems was 1.5 µs.

### Statistical analysis

For the MTT assay, six replicate tests were carried out for each concentration. Data are presented as mean and SD. Two-way ANOVA was used to analyze the difference between treated and control groups in the Origin program. *P* value, *P* < 0.001 was considered statically significant.

## Results and discussion

### Characterization of GO nanoparticles

The GO nanosheets were synthesized by modified Hummer’s method^38,39^. The XRD pattern of GO nanosheets is shown in Fig. S3. The d-spacing of GO is higher than the graphene. This increase in spacing between the graphene layers is related to the incorporation of oxidized surface groups and confirm the oxidation of the graphene^40–42^. The FTIR spectroscopy was carried out to characterize the functional groups in the GO sample. The broad peak observed at 2900–3500 cm^−1^ is related to O–H vibrations for GO nanosheets that reveal the presence of the hydroxyl groups in the GO nanosheet. These O–H groups are bonded to a carbon network at various positions in nanosheets. A peak at 1630 cm^−1^ is related to the vibrations of adsorbed water molecules. The sharp peak at 1630 cm^−1^ can be assigned to the stretching and bending vibration of OH groups of water molecules adsorbed on GO. The basic component of graphene was approved by an absorption peak of C = C at ~ 1575 cm^−1^ which is related to the phenol C = C ring stretching and shows some sp2 C = C bonds are un-oxidized. In addition, a peak at 1023 cm^−1^ is related to C = O bonds. The peak at 1225 cm^−1^ is related to the C–O–C stretching and the peak at 1056 cm^−1^ corresponds to the vibrational mode of the C-O group^39,43^ (Fig. S3).

The UV–Vis absorption spectra of GO nanosheets are shown in Fig. S4**.** The GO has a main absorption peak at 230 nm and a shoulder peak at 300 nm which is related to the π-π* transitions C=C bond and n-π* transitions of C=O bond^44,45^ (Fig. S4). Moreover, the absorption of GO solution at different concentrations is shown in Fig. S4, and the relationship between the absorbance intensity and the concentrations of GO solution traced in Fig. S4 which confirmed with previous studies^46,47^ (Fig. S4).

The prepared GO nanosheets were characterized by Raman spectra. Raman spectroscopy is a non-destructive method to characterize the electronic structures of the carbon nanostructure such as crystal disorder, degree of hybridization, and chemical modification^48,49^. The Raman spectra of GO showed a G band related to first-order scattering around 1360 cm^−1^ and a D band arising from the doubly resonant disorder-induced mode around 1600 cm^−1^ which is consistent with previous results^50,51^. The ratio of the intensity of I_D_ / I_G_ for GO nanosheets is 1.1 (Fig. S5). The high value of the D peak in this ratio shows the significant structural disorder due to the oxygen functional groups. The G peak of GO is shifted to higher energy and widened related to the graphite which shows the decrease in the in-plane crystal and conversion of sp^2^ to sp^3^ carbon bonds^52^.

The shape and position of the 2D bond of GO in Raman spectra around 2780 cm^−1^ shows the GO sample has few layers^53^ (Fig. S5). The feature of the 2D peak provides information about the quality of graphene oxide. The low intensity and broad 2D peak for GO relate to graphene indicates the steric effects of oxygen groups on layers^51^.

Atomic force microscopy was applied to characterize the morphology and thickness of the GO. The height profile of GO nanosheets shows that the thickness of GO nanosheet is around 1.7 nm. The length of the GO sheet is around 100–200 nm calculated by atomic force microscopy. The GO sheet has a negative charge in PBS solution, confirmed by − 30.36 ± 1.0 value of Zeta potential. Analysis of DLS results indicated that the hydrodynamic size of the GO sheets is about 80–100 nm. These results were consistent with the results of previous work (Fig. S2)^13,26^.

### Cell viability analysis in the microfluidic chip

**Figure 1:**
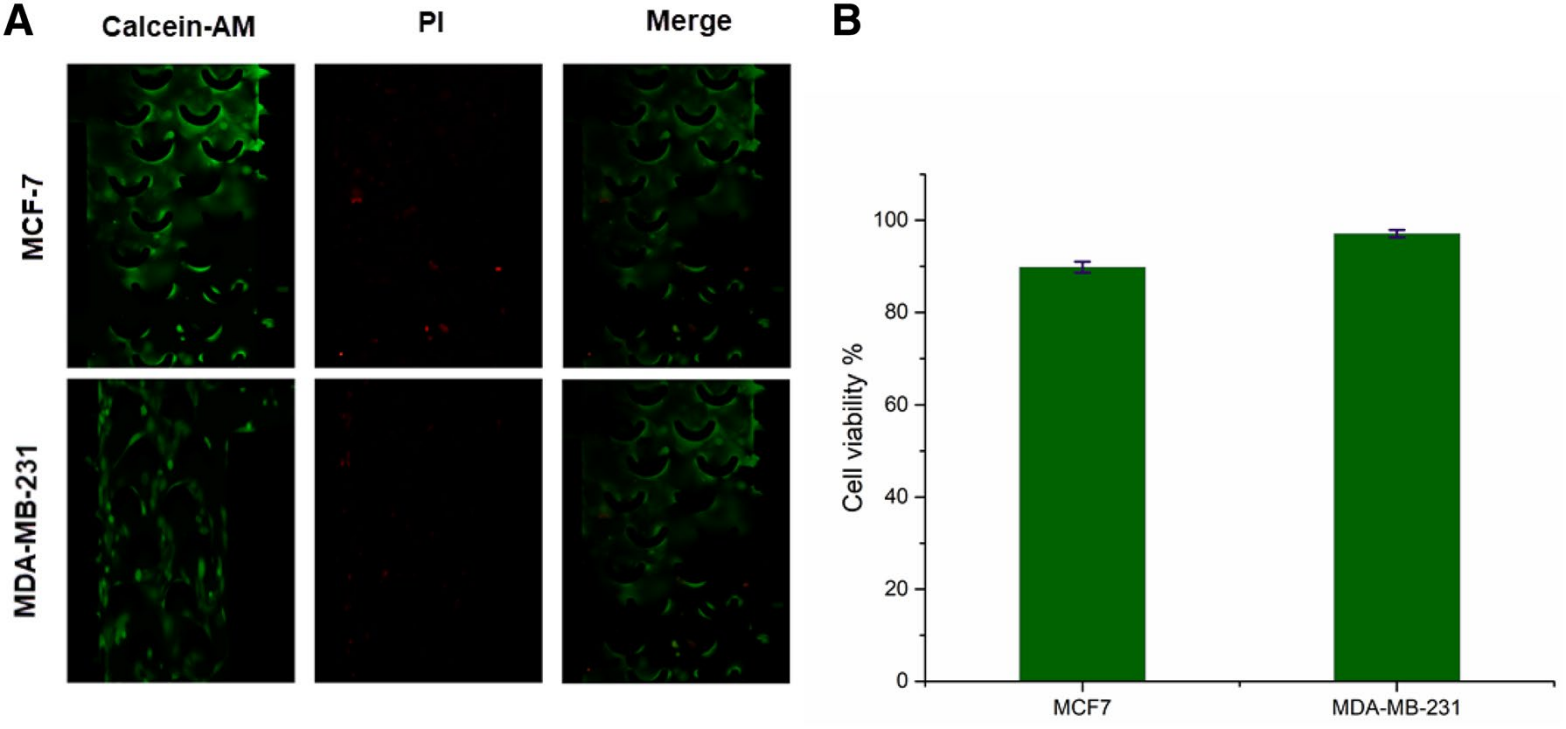
(**A**) Fluorescence images of live/dead assay, the green color shows the live cells and red color shows dead cells of MDA-MB-231 and MCF-7 cell lines (**B**) cell viability of the MDA-MB-231 (96.4%) and MCF-7 (91.3%), the error bar show the standard deviation (n = 4).

In order to observe cell viability on a microfluidic chip, MDA-MB-231 and MCF-7 cell lines were loaded into the main chamber of microfluidic chips and analyzed the cell viability by using fluorescence images at 480 nm and 590 nm, in which the green and red showed viable and dead cells, respectively. All of the images were taken 48 h after cell loading in the main chamber of microfluidic devices (Fig. 1A). Our results show that the viability of both cell lines was above 90% (Fig. 1B).

### The effect of GO on migration properties of cancer cells

**Figure 2:**
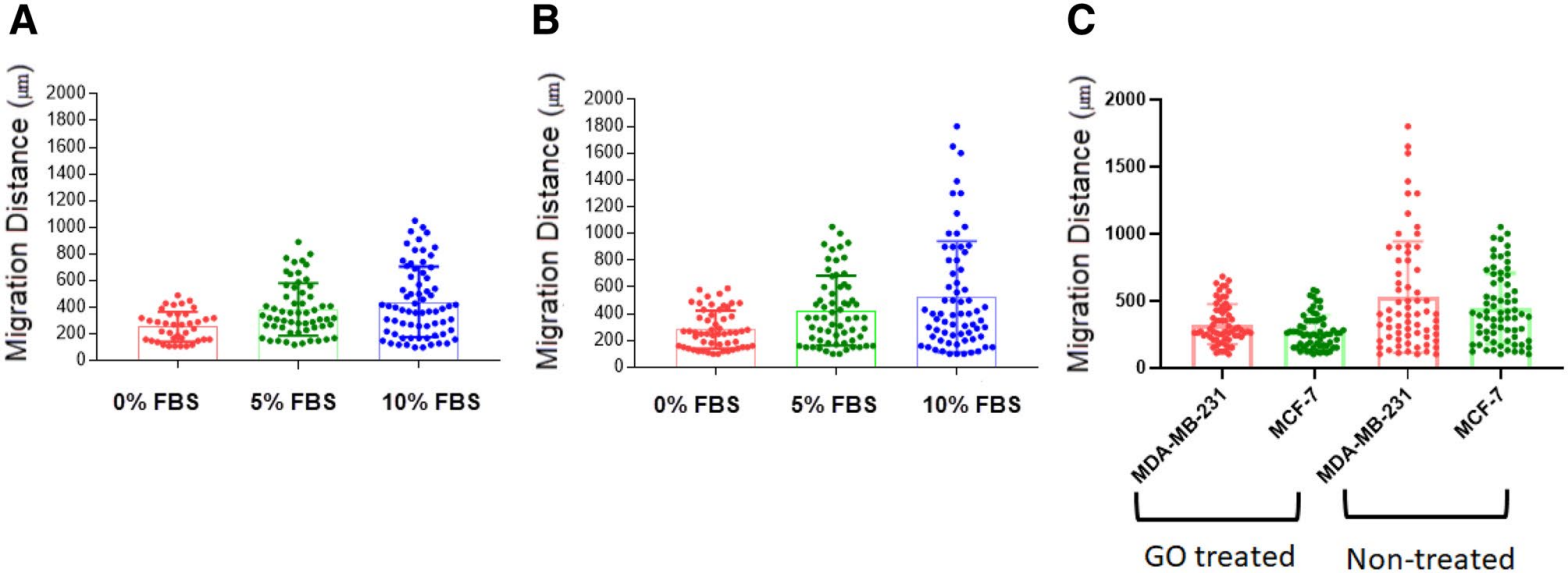
(**A**) The box plot of cell migration of MCF-7 and (**B**) MDA-MB-231 cell lines and (**C**) The cell migration of both cell lines in untreated and treated by the GO nanosheets. Each spot represents one cell in a microfluidic chip, which was obtained from five different independent experiments.

The migration properties and epithelial-mesenchymal transition are the key parameters in cancer progression, inflammation, wound healing, and angiogenesis^24,54–56^. We investigated the effect of GO on cell migration. We have prepared a microfluidic device to investigate the migration properties. These results showed that GO can inhibit migration in MCF7 and MDA-MB-231 cell lines significantly (Fig. 2). The cancer cells can be moved by chemoattractant compounds and the gradient of growth factor or other chemoattractant compounds could induce migration of cancer cells in the direction of the gradient of chemoattractant agents^57–59^. The microfluidic chip has a main chamber for cell loading and trapping and also 100 miniaturized chambers for cell migration using this microfluidic chip. We have obtained two key quantitative parameters in cell migration including the velocity of cells and cell heterogeneity at the single-cell level.

We studied the cell migration properties of breast cancer cells in the microfluidic chip. In the absence of chemoattractant stimulus, few MCF7 and MDA-MB-231 cells migrated through narrow channels. However, we observed a chemo-attractive response in 10% FBS as a chemoattractant stimulus^24^. Based on our results, there was a trend that the migration distance of cancer cells became larger with increased chemoattractant concentration in the microfluidic chip. In treated cells by 10 µg mL^−1^ GO, we observed 39.2% and 38.6% inhibition mesenchymal migration for MCF7 and MDA-MB-231 cell lines compared to control cancer cell (Fig. 2A,B). In the MCF7 cell line, the mean velocity migration for untreated and treated cancer cells was measured as 18.4 µm/h and 11.2 µm/h respectively (Fig. 2A,B). In the MDA-MB-231 cell line, the mean velocity migration for untreated and treated was calculated as 22.1 µm/h and 13.5 µm/h respectively. The mean velocity of untreated cancer cells is higher compared to treated cancer cells (Fig. 2C). Our results showed that GO nanosheets can be reduced the velocity of cell migration compared to control cells, however, the GO nanosheet treated did not induce a cytotoxicity effect on cancer cells. In addition, it has been reported that actin-bundling has an important role in cellular properties such as cell polarization, cell migration, and movement^60,61^. Therefore, the actin polymerization and shapes, and orientation of actin filaments can highly affect cell migration^1^. This suggests that migration inhibition of GO nanosheets is related to interactions between GO nano-sheets and cytoskeleton in cancer cells.

### Cytotoxicity of the GO nanosheets against the MCF-7 and MDA-MB-231 cell lines

**Figure 3:**
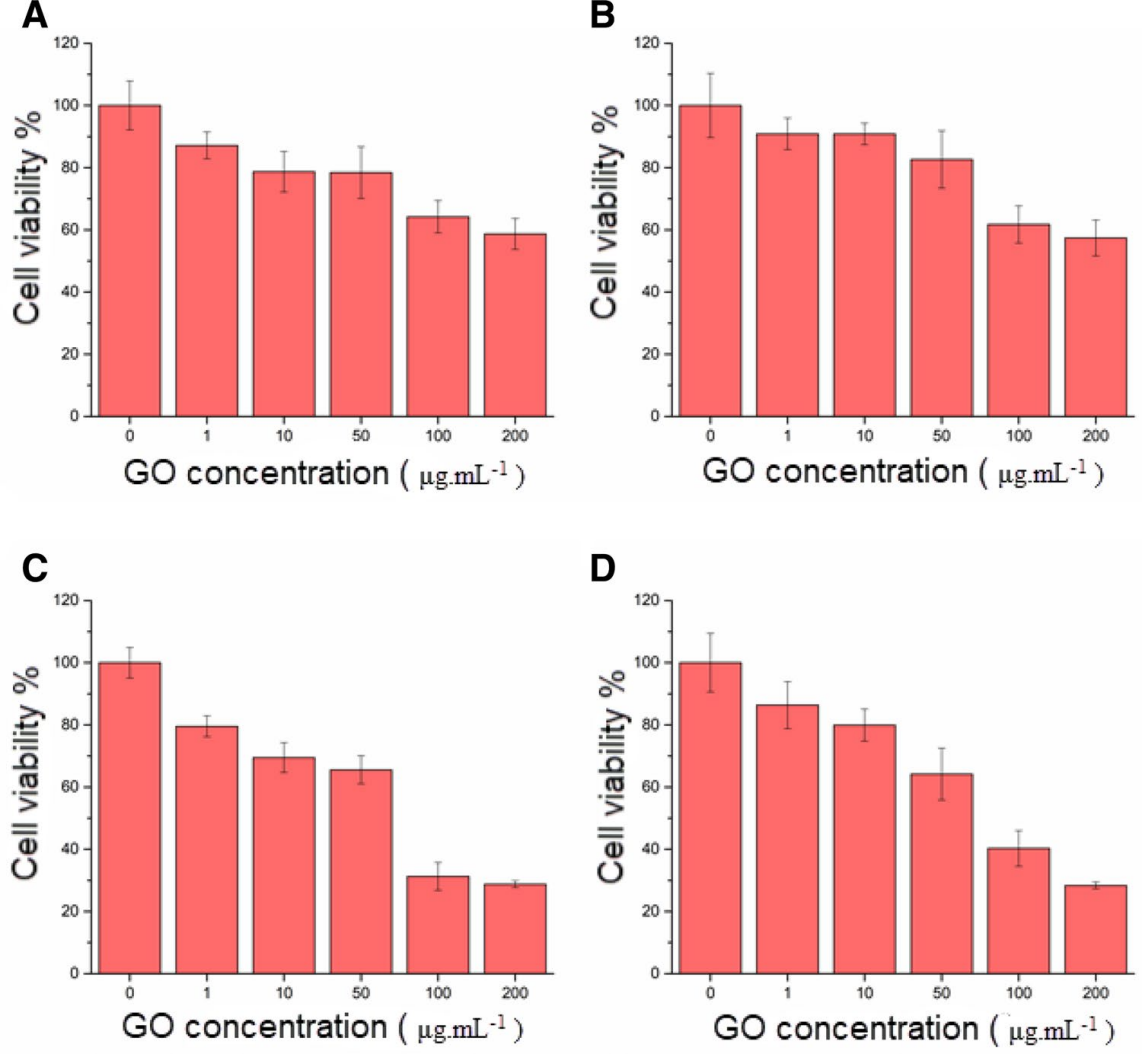
(**A**) The cell viability of MCF-7 cell line after GO nanosheet treated 24 h (**A**) and 48 h (**C**) and the cell viability of MDA-MB-231 cell line after GO nanosheets treated 48 h (**B**) and 24 h (**D**), The data represent the mean ± standard deviation of cell viability of MCF-7 and MDA-MB-231 cell lines obtained from four independent experiments.

In order to investigate the cytotoxicity of the GO nanosheets on cells, an MTT assay was carried out. We observed reduced viability at high concentrations (100 and 200 µg mL^−1^) of the GO nanosheets in the MCF-7 and MDA-MB-231 cell lines. For low concentrations of the GO nanosheets (1, 10, 50 µg mL^−1^), cell viability was above 80% for MDA-MB-231 and MCF-7 cell lines (Fig. 3). Our results revealed that the three tested concentrations (1, 10, 50 µg mL^−1^) of the graphene oxide did not have significant cytotoxicity in both cell lines. Our cytotoxicity results of the GO nanosheets showed good consistency with the previous works^62^.

### Effect of the GO nanosheets on the stiffness of cancer cells

Biophysical features of cells play an important role in a large range of cellular processes. These features include cell morphology, fractal dimension, and cell stiffness. The stiffness is a key parameter in several physiological and pathological conditions^63–66^. It has been reported that the stiffness of cells significantly changes during cancer progression^23,67^. In addition, softer cells might have higher metastatic and migration properties. Therefore, cell stiffness is a research topic for the diagnostic and therapeutic methods for cancer^12,23,68^. In order to monitor the effect of the GO nanosheets on the cell stiffness, this parameter for MDA-MB-231 and MCF7 cell lines was measured in different concentrations of the GO nanosheets. Our results showed that the cell stiffness in MCF7 treated with the GO sheet significantly decreased whereas the stiffness of the MDA-MB-231 cell line slightly decreased. The cell stiffness of MCF7 and MDA-MB-231 cell line is about 2.44 kPa and 1.32 kPa which is the lower stiffness value for the MDA-MB-231 cell line related to the higher metastatic properties.

**Figure 4:**
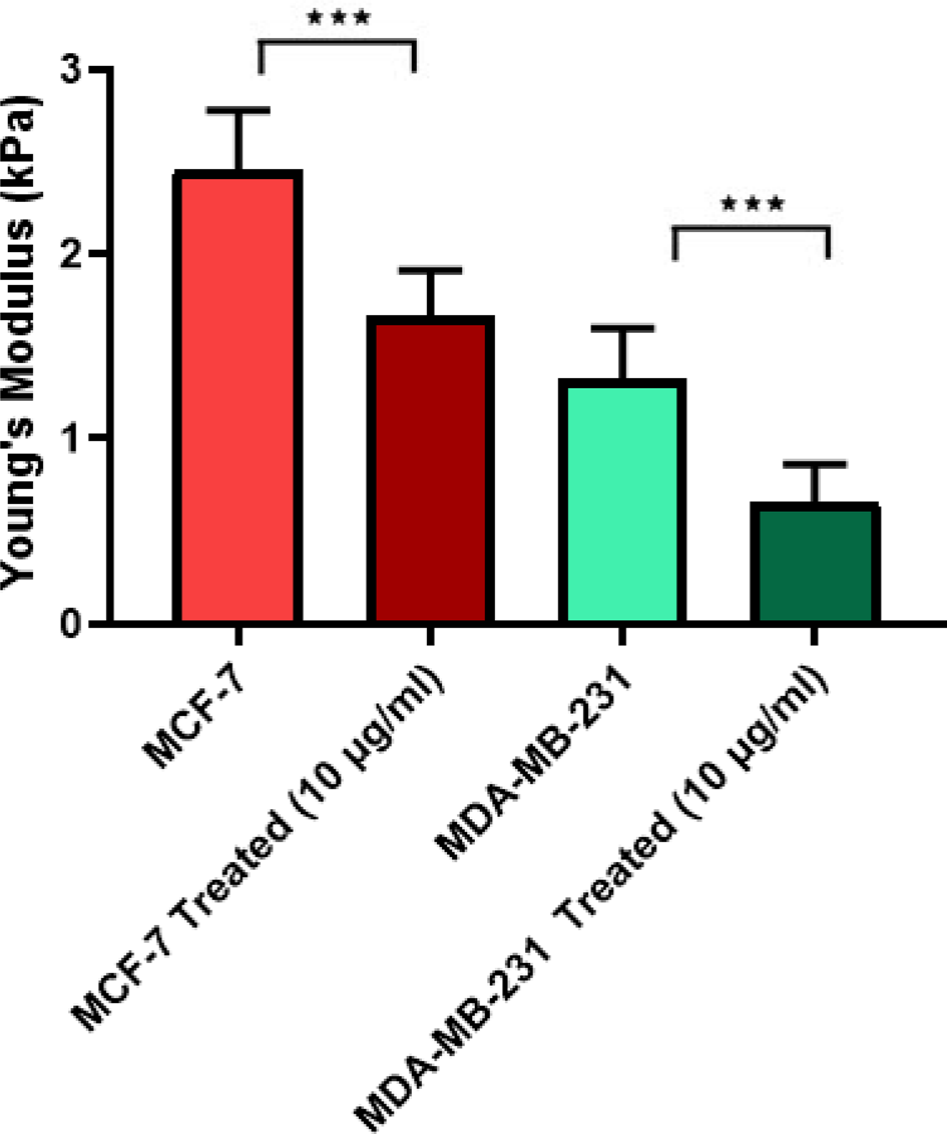
The effect of GO nanosheets Young’s modulus of MCF-7 and MDA-MB-231 cell lines. The data represent the mean ± standard deviation of Young’s modulus of MCF-7 and MDA-MB-231 cell lines obtained from six independent experiments. Two-way ANOVA was used to analyze the difference between treated and control groups and *P < 0.05, **P < 0.01, ***P < 0.001, and ****P < 0.0001.

The Young’s Modulus of the MCF7 and MDA-MB-231 were about 2.44 kPa and 1.32 kPa respectively (Fig. 4). Compare to the control group, we observed a significant reduction in the cell stiffness (from 2.44 kPa to 1.65 kPa) in the MCF7 cells treated by the GO. In the MDA-MB-231 cell line, reduction in the cell stiffness is highly related to the MCF7 (from 1.32 kPa to 0.65 kPa) (Fig. 4). Our results show that the GO nanosheets can induce significant changes in the stiffness of cells without cytotoxic effect on them. It seems that the GO nanosheets can induce changes in biomechanical characteristics such as the cell stiffness and migration properties of cells due to their effects on the intracellular cytoskeleton.

### Effect of the GO nanosheet on the actin cytoskeleton

**Figure 5:**
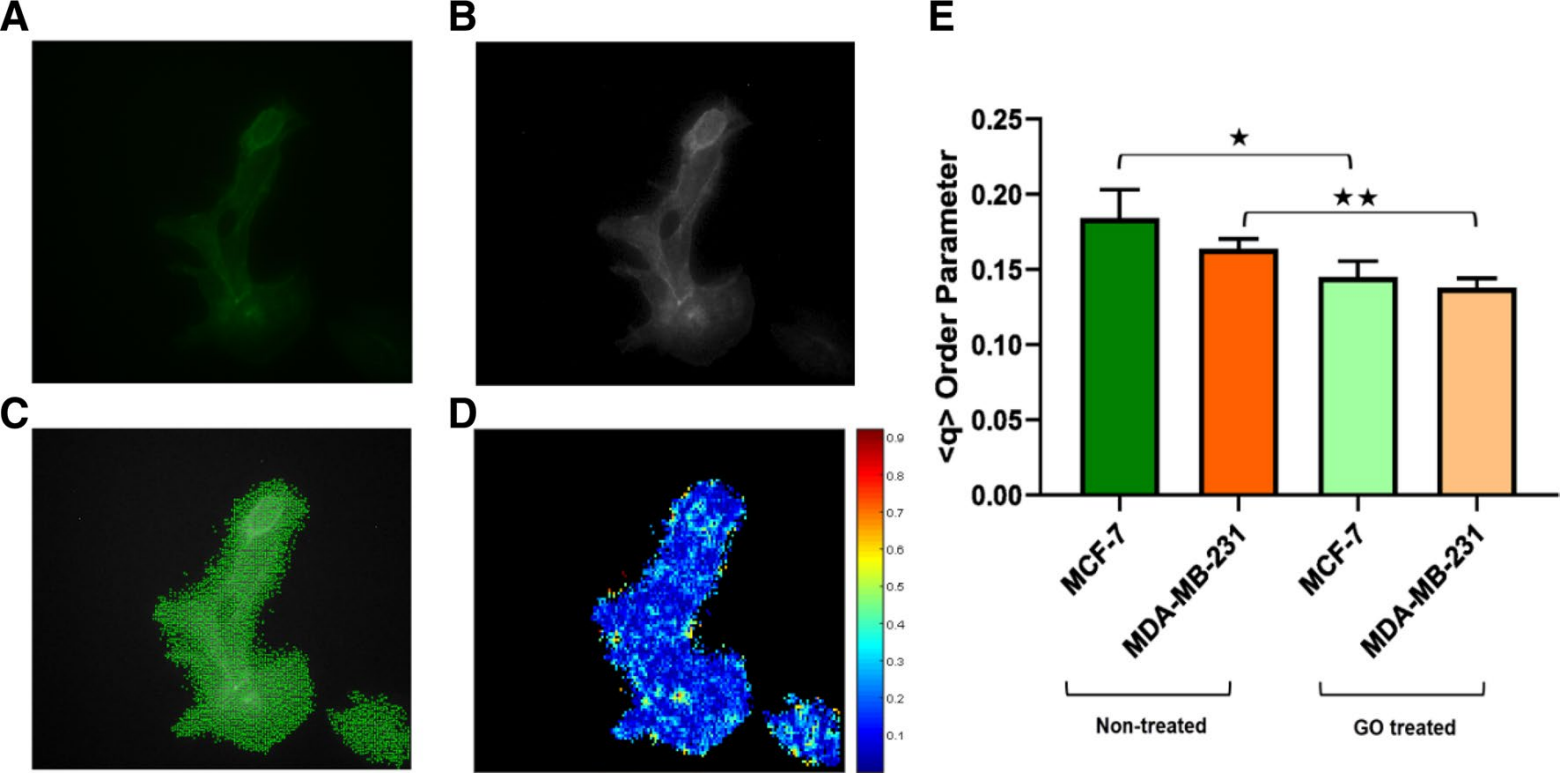
Effect of GO nanosheet on F-actin order parameter in MDA-MB-231 and MCF-7 cell lines. (**A**) Fluorescence F-actin network images, (**B**) and the masked image of fluorescence image, (**C**) The local orientation of actin filaments in the fluorescence image, (**D**) Heat map of order parameter < q > for MDA-MB-231 and MCF-7 cell lines non-treated and GO nanosheet treated, (**E**) The statistical analysis of the effect of GO on average of the order parameter of actin filaments in MCF-7 and MDA-MB-231 cell lines. The data represent the mean ± standard deviation of the order parameter of MCF-7 and MDA-MB-231 cell lines were obtained from three independent experiments. Two-way ANOVA was used to analyze the difference between treated and control groups and *P < 0.05, **P < 0.01, ***P < 0.001, and ****P < 0.0001.

Previous studies showed that there is a relationship between the orientation of the cytoskeleton and mechanical properties in cells. The cytoskeletal orientation of cells plays a key role in the mechanical and biochemical properties of cells^11,69^. In order to investigate the effect of the GO nanosheet on the cytoskeleton orientation of cells, the actins network was stained and mechanical parameters were derived from these images. The changes of the actin filaments organization were quantified by an order parameter $q = 2\left( {cos^{2} \left( \theta \right) - \frac{1}{2}} \right)$, where the image was segmented to the number of overlapping blocks, then the local actin orientation (director) was calculated for each block, and $\theta$ is the angle difference between the directors of the central block and surrounding blocks (Code used to measure the nematic order parameter is available at: https://github.com/OakesLab/FFT_Alignment.)^70,71^. $q$ ranges from zero to one which is showing randomly oriented directors and parallel directors respectively. The results showed that the GO nanosheet slightly affects the order parameter related to Ctrl conditions of MDA-MB-231 and MCF-7 cell lines (Fig. 5D). The average of < q > of untreated and treated MCF-7 was 0.189 and 0.145 (Fig. 5E). Moreover, the averages of < q > of MDA-MB-231 untreated and treated were 0.176 and 0.140 (Fig. 5E). Our results indicated that the GO nanosheet can decrease < q > in actins network in both cell lines, especially in the MCF-7 cell line.

The order parameter of the actin filaments has a key role in the mechanical properties of cells and a positive correlation between < q > parameter of the actin filaments and the mechanical properties of the cells have been reported^11,70^. In our study, we established a quantitative picture of the mechanical properties of the cell’s responses to the nanoparticles. Our study also, reveals that the GO nanosheets can change < q > parameter of the actin filaments since the GO nanosheet directly binds to the actin filaments and changes the actins network parameters. In the presence of the GO nanosheets, the < q > at the cellular level leads to the lower actin order parameter and the cell stiffness. Previous reports by Lee and Tojkander showed that there is a correlation between cell stiffness and actin filament orientation^12,72^. It has been reported that the lower stiffness of cancer cells in breast cancer is related to the disruption of well-organized actin filaments which play a key role in the mechanical cell properties^12,72^. It seems that the GO nanosheet can play a key role in changing the cytoskeleton organization in cells which leads to altering the mechanical parameters of cells such as the cell stiffness.

As hypothesized, the GO nanosheets can change the mechanical properties of cells. The mechanical properties of cells are highly regulated by several intracellular processes and any changes could result in fatal consequences^73^. The key elements that regulate the mechanical properties of the cells such as stiffness and shape are the cell's cytoskeleton and the physical properties of the environment. Studies have shown the cytoskeleton of cells is altered by drugs or nanoparticle treatment such as cisplatin and fluorene^1,74^. Our results have shown that the GO nanosheets can change the cytoskeleton filaments’ organization. Our finding showed the reduction of the order parameter in actin filaments was 23% in the MCF7 cell line and 20.4% in the MDA-MB-231 cell line (Fig. 5). The cytoskeleton organization changes are able to differ the cell stiffness. Cell motility is a physical property of the cells, which has critical roles in a variety of cell processes such as cell migration, invasion, and angiogenesis processes. Studies have shown that the motility of cells is related to the cytoskeleton organization. The cytoskeleton organization changes can lead to alteration of cell motility properties which results in fatal consequences. Therefore, the GO nanosheets can change the structure of the actin filaments and reduce the motility of cells.

Moreover, the F-actin intensity for all cells was calculated by ImageJ software. There were no significant differences between the GO treated and untreated conditions.

### Coarse-grained simulation of the actin–GO interaction

**Figure 6:**
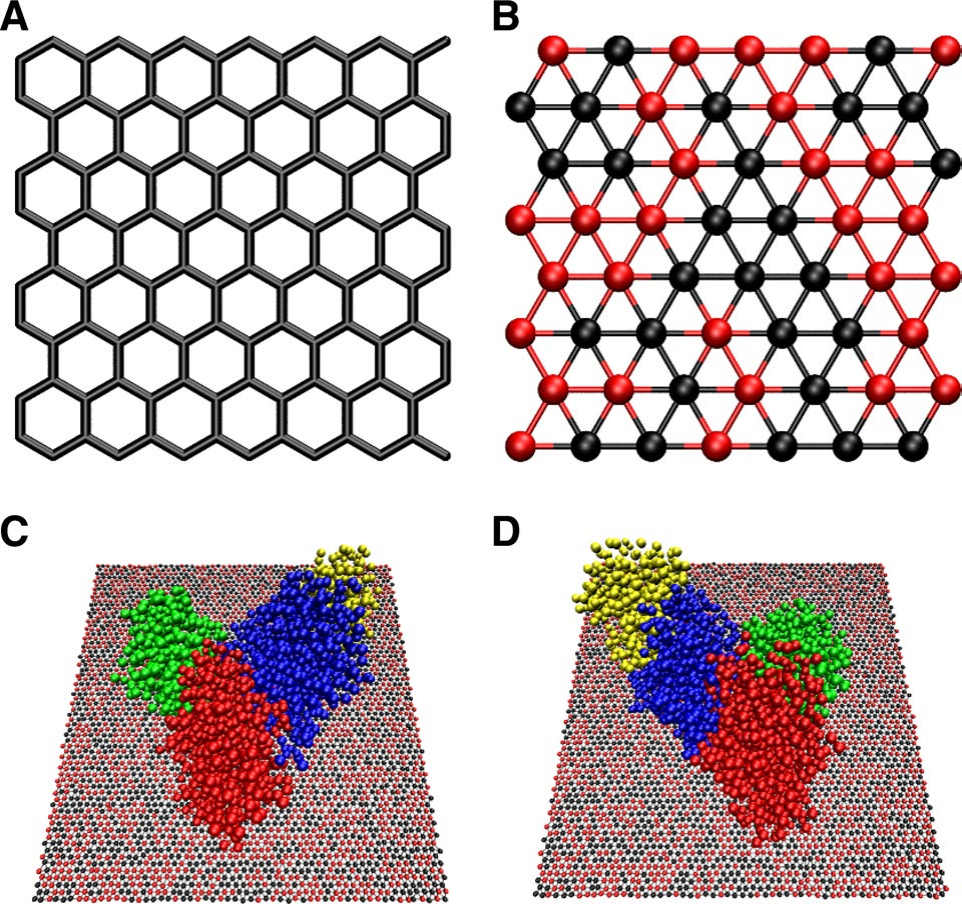
The atomistic structure of graphene (**A**) and coarse-grained structure of GO nanosheets (**B**) and images of (**C**) and (**D**) show the first and second orientations of GO nanosheets and actin filament.

MD simulation is a powerful tool for the study of biomolecules’ nanomaterial interaction in atomic details^75,76^. In order to understand the biomolecules–GO interactions, the MD simulation was used to observe these interactions and study how the GO nanosheets change the structures of actin polymer. In Fig. 6A,B, we showed the atomistic structure of graphene and coarse-grained structures of the GO nanosheets. Both simulation systems were shown in Fig. 6C,D.

**Figure 7:**
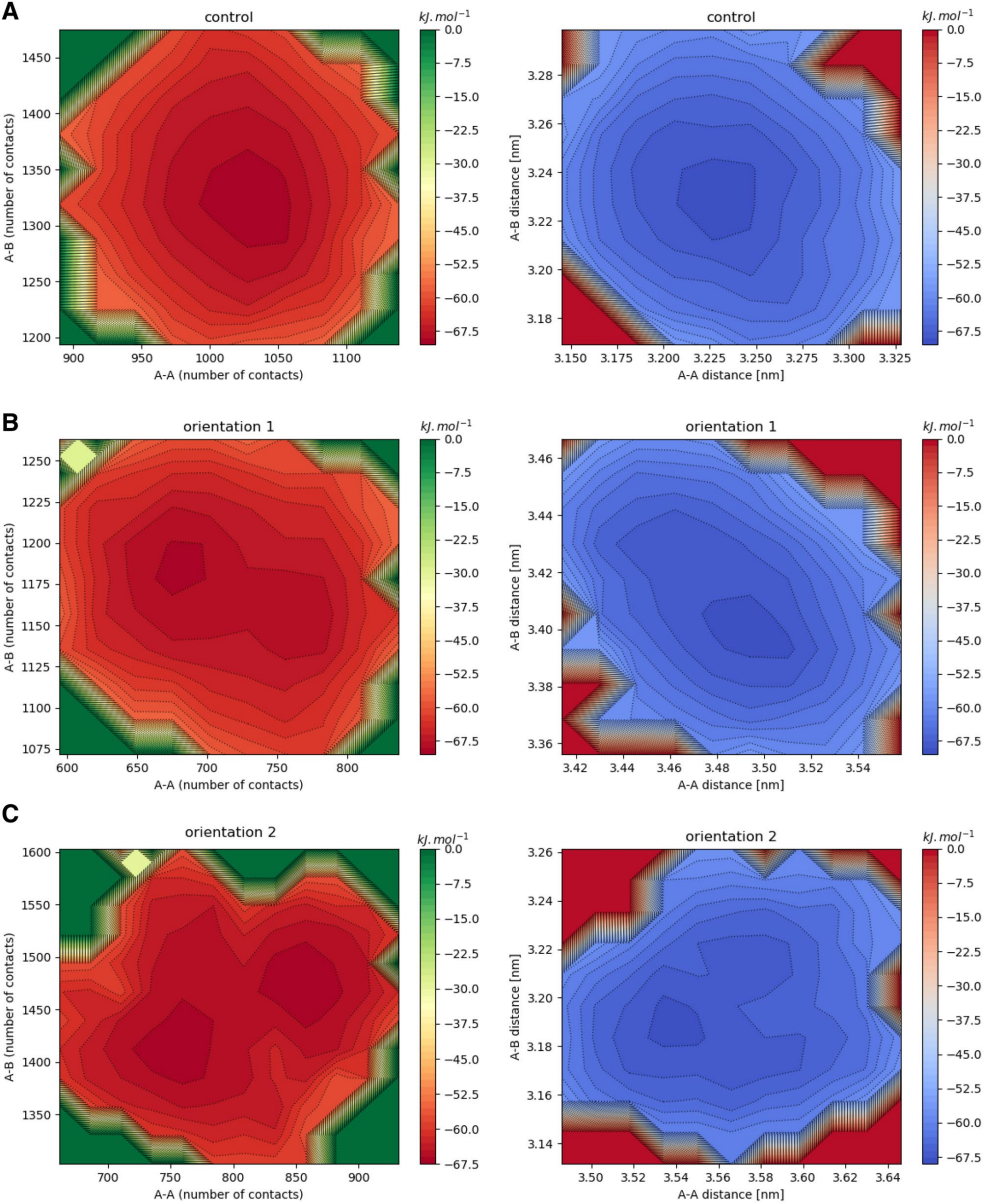
The 2D free energy plots. (**A**–**C**) The 2D free energy plots of distance actin-actin and actin-cofilin and number of contact actin-actin and actin-cofilin for control, orientation1, and orientation2. In this plot, actin-actin and actin-cofilin were shown by A–A and A–B.

To monitor the actin disassociation of the actin filament during MD simulation, the distances between the center of mass (COM) actin–actin subunit and actin–cofilin were calculated. Moreover, the changes in actin–actin, and actin–cofilin interactions upon the GO interaction were monitored by calculating the total native contacts. To assess the conformational space of our systems, we plotted the 2D free energy landscape as a function of native contacts and distance between the center of mass (COM) of actin–actin, and actin–cofilin. Our results showed the distinct minima along with R A-A (actin-actin) and R A-B (actin-cofilin) for the actin filament without the GO nanosheets located around (3.22 nm, 3.23 nm), whereas in the presence of the GO, distinct minima for orientation 1 and orientation 2 located at (3.5 nm, 3.4 nm) and (3.5 nm, 3.3 nm) (Fig. 7B,C). These results indicated that the GO could slightly increase the distance between actin subunits and also the GO nanosheet can dissociate actin subunits from each other. In 2D free energy landscape of actin–actin, and actin–cofilin contacts, one distinct minima changes in two distinct minima in oreintation2 and large changes in free energy plot for oritentation1. The results show that in the actin filament without the GO, there was a distinct minimum located at (1050, 1100), whereas the free energy landscape of both the actin–GO systems have large changes related to the control condition (Fig. 7A). In system orientation1, there were distinct minima located at (650, 1100) (Fig. 7B). In system orientation2, two distinct minima were found in 2D free energy landscape which located at (750, 1300) and (850, 1400) (Fig. 7C). These results show that the native contact between the actin–actin decreased upon the GO binding. The GO nanosheets have several targets in intracellular components such as the actin filaments which determines the mechanical cell elasticity. The actins are organized into different structures that play a critical role within cells. Additionally, several pieces of evidence showed the GO can enter cells and directly bind to actin filaments^72^.

Our finding showed that the GO nanosheets can directly bound with the actin filaments and lead to changes in structures of actin filaments.

The analysis of 2D free energy plots shows that the GO nanosheet can induce large changes in the actin filaments. It seems that the GO nanosheet could significantly increase distances of the actin-actin subunits and disrupted the actin filaments.

**Figure 8:**
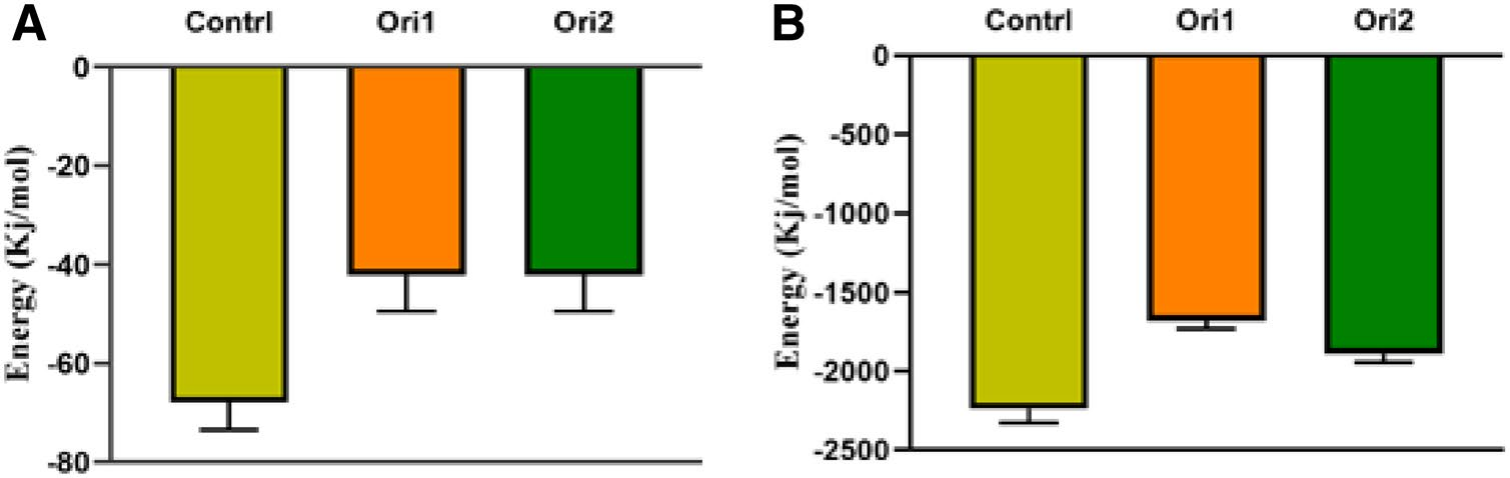
The VdW and electrostatic energies for control, orientation1, and orienation2 systems during the last 300 ns of MD simulations.

The VdW and electrostatic energy between the actin-actin subunits indicate that GO nanosheet can changes interactions between two subunits. The VdW and electrostatic energy between the actin-actin filaments for control were 2250 ± 50 and 69 ± 7 kJ mol^−1^ (Fig. 8A,B). While the VdW energy for the orientation1 and orientation2 were 1740 ± 60 and 1950 ± 40 kJ mol^−1^ and electrostatic energy for the orientation1 and orientation2 were 41 ± 4 and 41 ± 5 kJ mol^−1^ respectively (Fig. 8A,B). It seems that the GO can change the VdW and electrostatic energies between actin subunits and in the following disrupt the actin subunits from each other in actin filament. Therefore, the GO can change the biological properties of cells due to direct binding to actin filaments and disrupting these structures.

## Conclusion

In this study, we investigated the biological effect of the GO nanosheets on two breast cell lines (MCF-7 and MDA-MB-231). Our finding indicated that the GO nanosheets could inhibit cell migration (39.2%) in MCF-7 and (38.6%) in MDA-MB-231 cell lines without causing cell death. In addition, we elucidated the GO could change the mechanical properties of cells such as the cell stiffness and the order parameter of the intracellular cytoskeleton. Our result showed that the GO nanosheets can decrease the cell stiffness by 32.4% in MCF7 cells and 30.3% in MDA-MB-231 cell line and the reduction of the order parameter of the actin filaments were 23% in the MCF7 cell line and 20.4% in the MDA-MB-231 cell line. It seems that the GO nanosheets can change the mechanical properties of cells such as the cell stiffness, cell migration, invasive properties, and order parameter of the intracellular cytoskeleton.

The GO nanosheets have several targets in the intracellular components such as the actin filaments which determines the mechanical cell elasticity. The actins are organized into different structures that play a critical role within cells. Additionally, several shreds of evidence showed that the GO can enter cells and directly bind to the actin filaments. To explain the mechanism of this effect, we showed that the GO nanosheets could disrupt the actin filaments by direct binding to them. The theoretical results show that the GO nanosheets can dissociate actin monomers from each other. The 2D free energy plot results show that the GO nanosheets can induce an increase in distance of the actin-actin subunits in the actin filaments and a decrease in the number of native contacts of the actin-actin subunits. The analysis of the VdW and electrostatic interactions between two subunits shows that the VdW and electrostatic energy between the actin-actin subunits decreased from 2250 to 1750 kJ mol^−1^ and electrostatic energy 69 to 41 kJ mol^−1^ due to the binding of the GO nanosheets. Furthermore, the interaction between the GO nanosheets and the actin filaments was stabilized during MD simulation which it seems VdW interactions have a more important role in the GO nanosheets–actin filaments interaction. Therefore, the GO nanosheets can change the mechanical properties of cells such as cell stiffness and motility due to dissociating actin subunits in filaments and changes the actin filaments orientation without causing cell death.

These findings give us a new viewpoint for cancer therapy by examining the mechanical cell properties such as the cell stiffness, motility, and orientation of the cell cytoskeleton. The carbon nanomaterial can be applied in a wide range of medicals applications. Our results showed that carbon nanomaterials can induce changes in the biological properties of cells without causing cell death. Therefore, the understanding of the effect of these materials on the biological properties of cells and the interaction of these materials with cells may be helpful in broadening the usage of carbon nanomaterial in medical applications.

## Supporting information

The schematic illustrations of the microfluidic chip and process of migration assay, characterization of the GO nanosheets by AFM (atomic force microscope) and DLS (dynamic light scattering), the cell migration process in the microfluidic chip, the green color shows the live cells, the Bright field pictures of MCF-7 cells treated by GO (10 ng mL^−1^) which showed the distribution of GO nanosheets on cells.

## Supplementary Information

Supplementary Information.

## Abbreviations

GO: Graphene oxide
DMEM: Eagle’s minimal essential medium
FBS: Fetal bovine serum
AFM: Atomic force microscopy
DLS: Dynamic light scattering
2D: Two-dimension
DMSO: Dimethyl sulfoxide, MTT
LINCS: Linear constraint
MD: Molecular dynamics
PDB: Protein data bank
SPC: Simple point charge
PME: Particle mesh Ewald
SPC water: Simple point-charge water
CG: Coarse-grained
PDMS: Poly dimethyl siloxane
PBS: Phosphate-buffered saline
