# Supplementary material for: Microfluidic investigation of the effect of graphene oxide on mechanical properties of cell and actin cytoskeleton networks: experimental and theoretical approaches
Source: Sci Rep. 2021 Aug 10;11:16216. doi: 10.1038/s41598-021-95624-0 (PMC8355332; doi:10.1038/s41598-021-95624-0)
Supplement: Supplementary file 1 — Supplementary Information. [file 41598_2021_95624_MOESM1_ESM.docx]

**Supporting Information**

**Microfluidic Investigation of the Effect of Graphene Oxide on Mechanical Properties of Cell and Actin Cytoskeleton Networks: Experimental and Theoretical Approaches**

Mohammad Ghorbani **^†^**, Hossein Soleymani **^†^**, Hadi Hashemzadeh **^‡^**, Saeed Mortezazadeh **^†^**, Mosslim Sedghi **^†^**, Seyedehsamaneh Shojaeilangari **^§^**, Abdollah Allahverdi **^†^**, Hossein Naderi-Manesh **^†^**^,^ **^‡^**^*^

**^†^** *Department of Biophysics, Faculty of Biological Science, Tarbiat Modares University, 14115-154 Tehran, Iran*

**^‡^** *Department of Nanobiotechnology, Faculty of Biological Science, Tarbiat Modares University, 14115-154 Tehran, Iran*

**^§^** *Biomedical Engineering Group, Department of Electrical Engineering and Information Technology, Iranian Research Organization for Science and Technology (IROST), P.O. Box 33535111, Tehran, Iran*

EMAIL:

*Mohammad Ghorbani:* [*mohammad.ghorbani*](mailto:mohammad.ghorbani@modares.ac.ir)*. @modares.ac.ir*

*Hossein Soleymani:* [*hsoleymani@modares.ac.ir*](mailto:hsoleymani@modares.ac.ir)

*Hadi Hashemzadeh:* [*hadi.Hashemzadeh@modares.ac.ir*](mailto:hadi.Hashemzadeh@modares.ac.ir)

*Saeed Mortezazadeh:* [*s.mortezazadeh@modares.ac.ir*](mailto:s.mortezazadeh@modares.ac.ir)

*Mosslim Sedghi:* [mosslim.sedghi@*modares.ac.ir*](mailto:mosslim.sedghi@modares.ac.ir)

*Seyedehsamane Shojaeilangari:* [*s.shojaie@irost.ir*](mailto:s.shojaie@irost.ir)

*Abdollah Allahverdi:* [*a-allahverdi@modares.ac.ir*](mailto:a-allahverdi@modares.ac.ir)

*Corresponding Author: Hossein Naderi-Manesh:* [*naderman@modares.ac.ir*](mailto:naderman@modares.ac.ir)

**Table of Contents**

**1. Figure S1.** The schematic illustrations of microfluidic chip and process of migration assay.

**2. Figure S2.** Characterization of GO nanosheets by AFM (atomic force microscope) and DLS (dynamic light scattering).

**Figure S3.** Characterization of GO nanosheets. A) FTIR and B) XRD pattern of GO.

**Figure S4.** Characterization of GO nanosheets. A) UV–vis absorbance spectra of GO in water at different concentrations. B) linear relationship between the absorbance intensity and the concentration of GO in water.

**Figure S5.** Characterization of GO nanosheets, the Raman spectra of GO

**Figure S6.** The cell migration process in the microfluidic chip, the green color shows the live cells.

**Figure S7.** The Bright field pictures of MCF-7 cells treated by GO (10 µg.mL^-1^ ) showed the distribution of GO nanosheets on cells.

**Figure S8.** A) The graphic data of Young’s modulus of a region of the cell from the MCF7 cell line, B) The graphic data of Young’s modulus of a region of the cell from the MDA-MB-231 cell line, C) The Force-Distance curve of the cells from the MCF7 cell line, D) The Force-Distance curve of the cells from the MDA-MB-231 cell line, E) The picture of the cells from MCF7 cell line F) The schematic illustration of The Young’s modulus measurement by AFM.


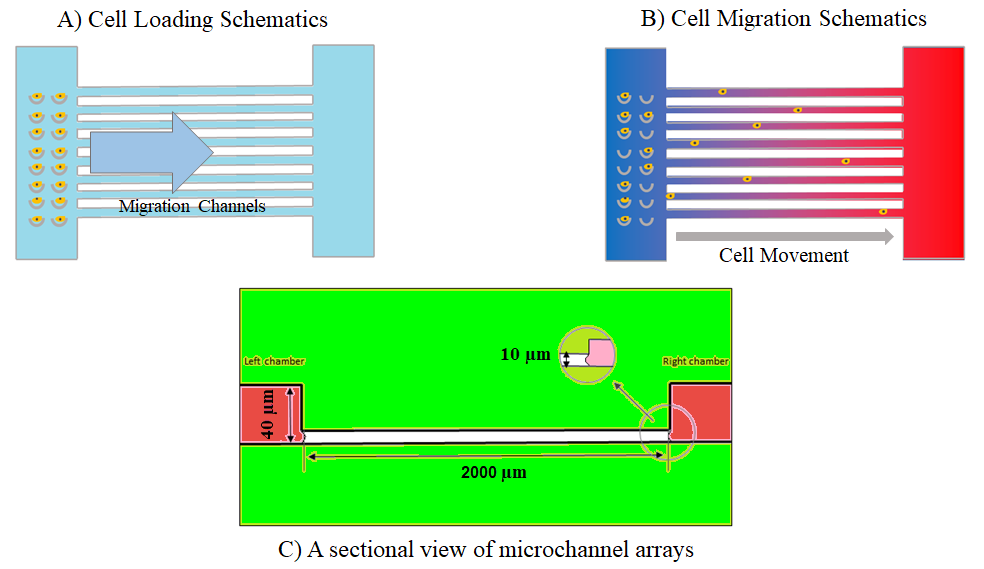


**Figure S1.** Schematic illustration of A) cell loading process, B) cell migration process, and C) a sectional view of microfluidic chip.

**
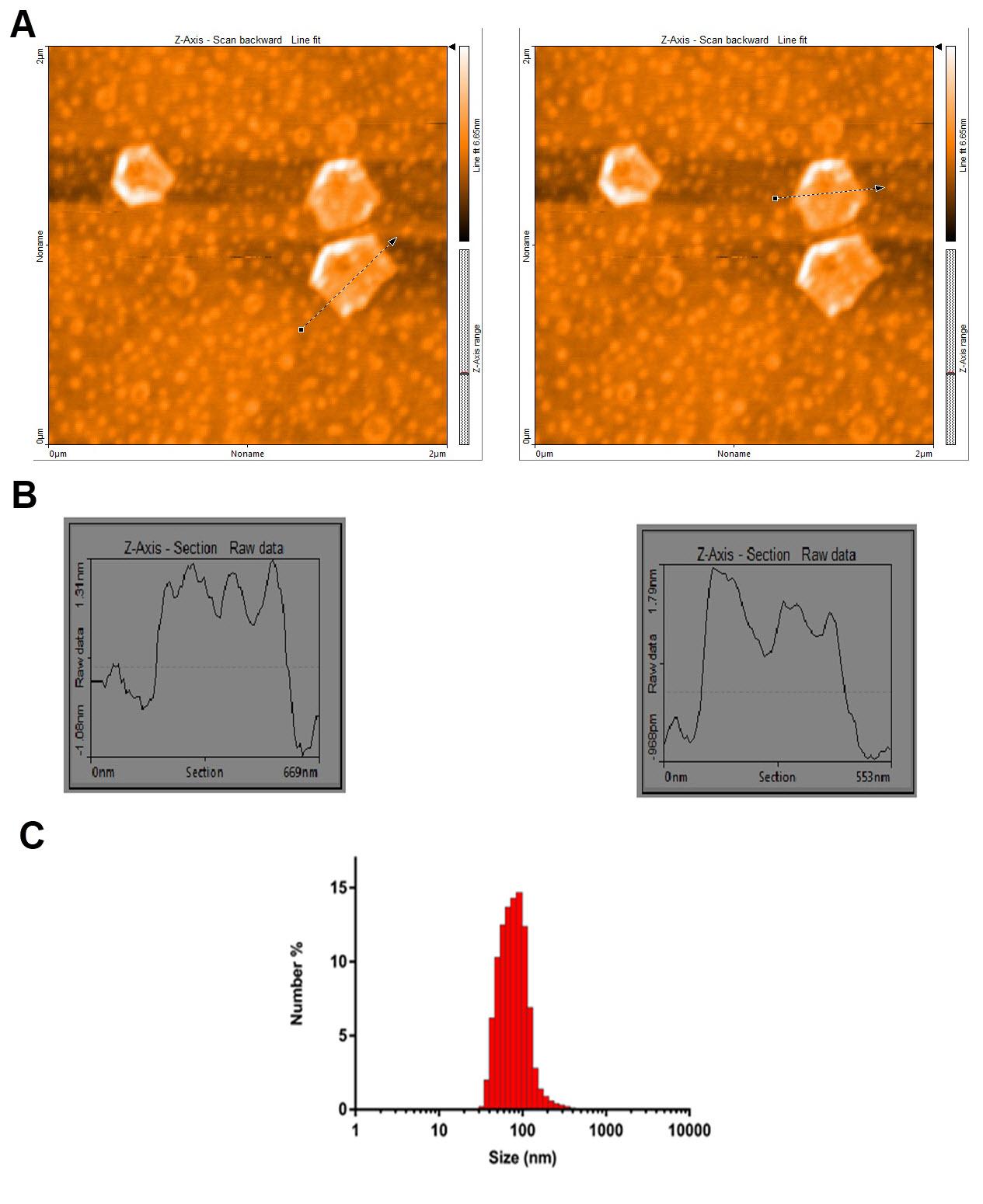
**

**Figure S2.** Characterization of GO nanosheets. A) The images of the GO nanosheets by AFM B) The height profile of GO nanosheets C) The size distribution of GO nanosheets.

**
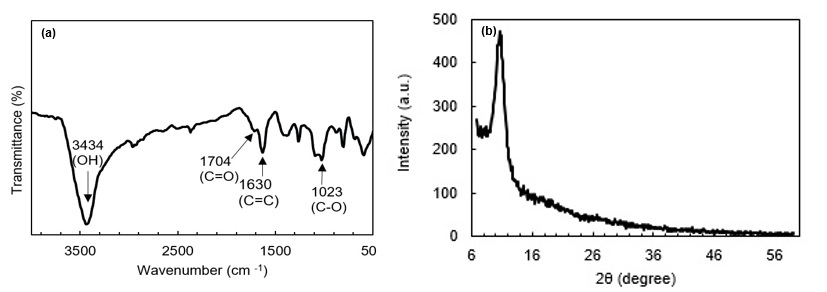
**

**Figure S3.** Characterization of GO nanosheets. A) FTIR and B) XRD pattern of GO.[[1](#_ENREF_1)]

**
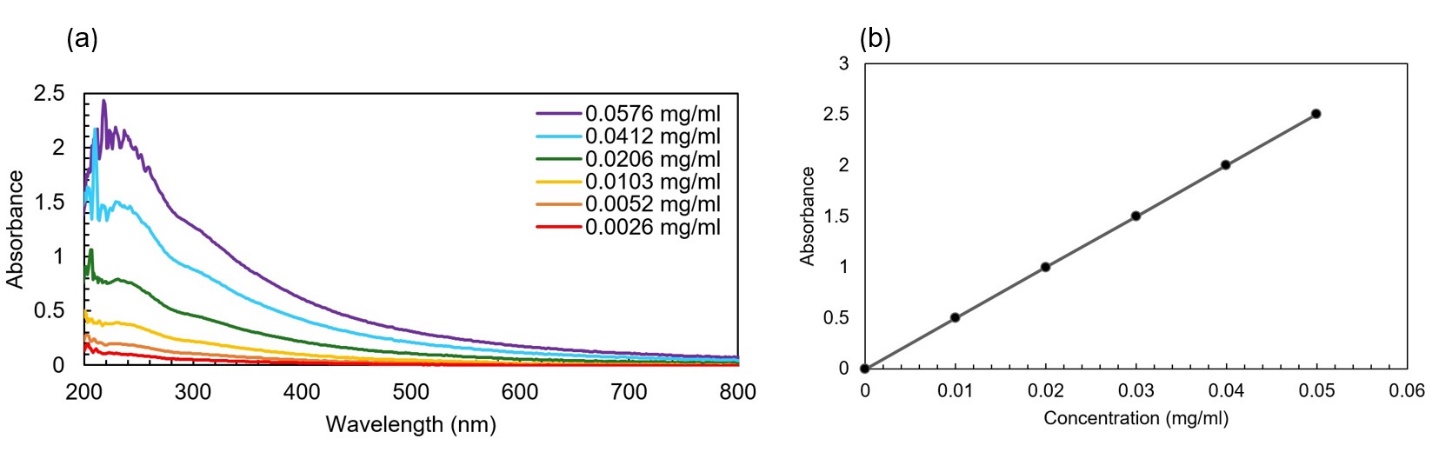
**

**Figure S4.** Characterization of GO nanosheets. A) UV–vis absorbance spectra of GO in water at different concentrations. B) linear relationship between the absorbance intensity and the concentration of GO in water.[[1](#_ENREF_1)]


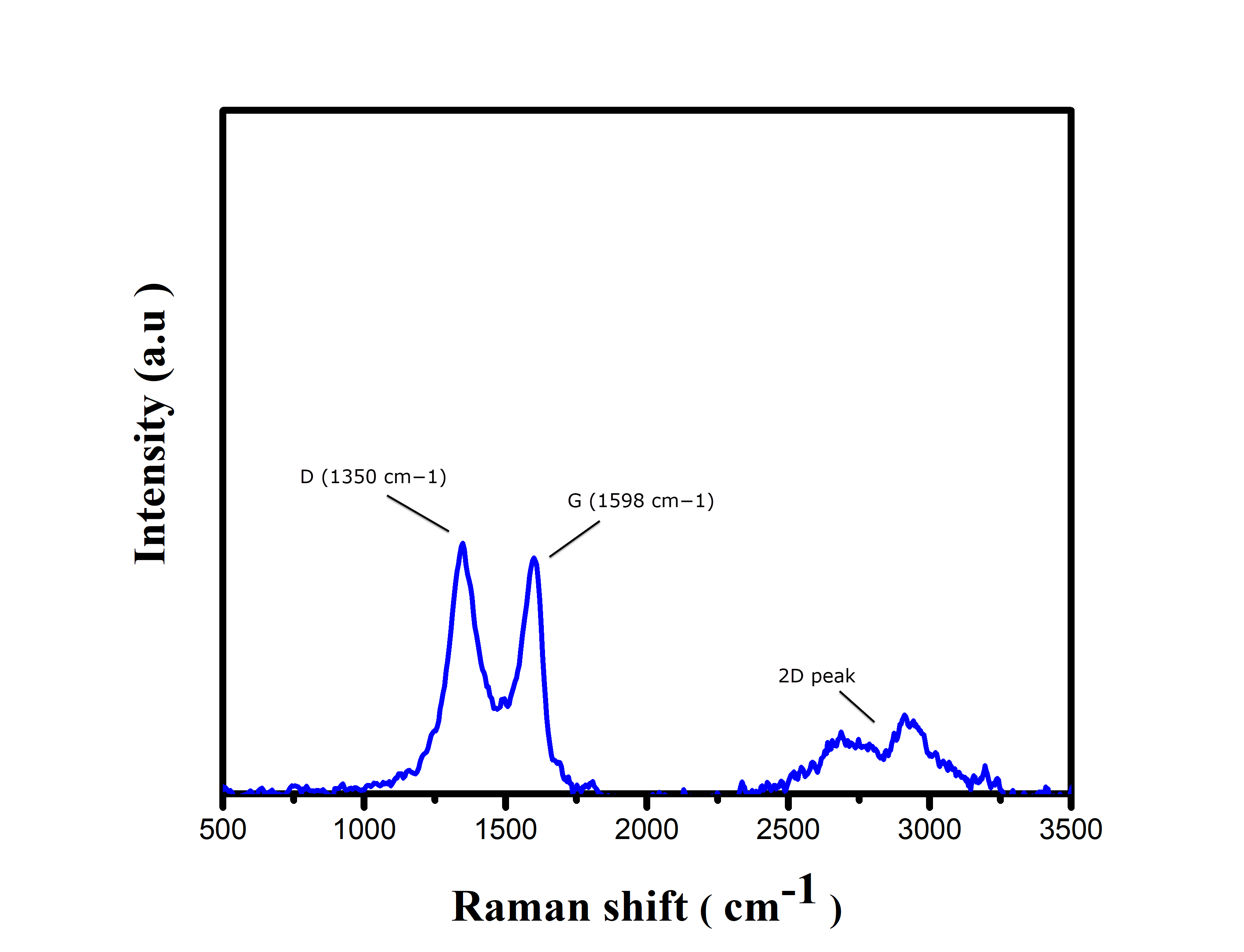


**Figure S5.** Characterization of GO nanosheets, the Raman spectra of GO.

**
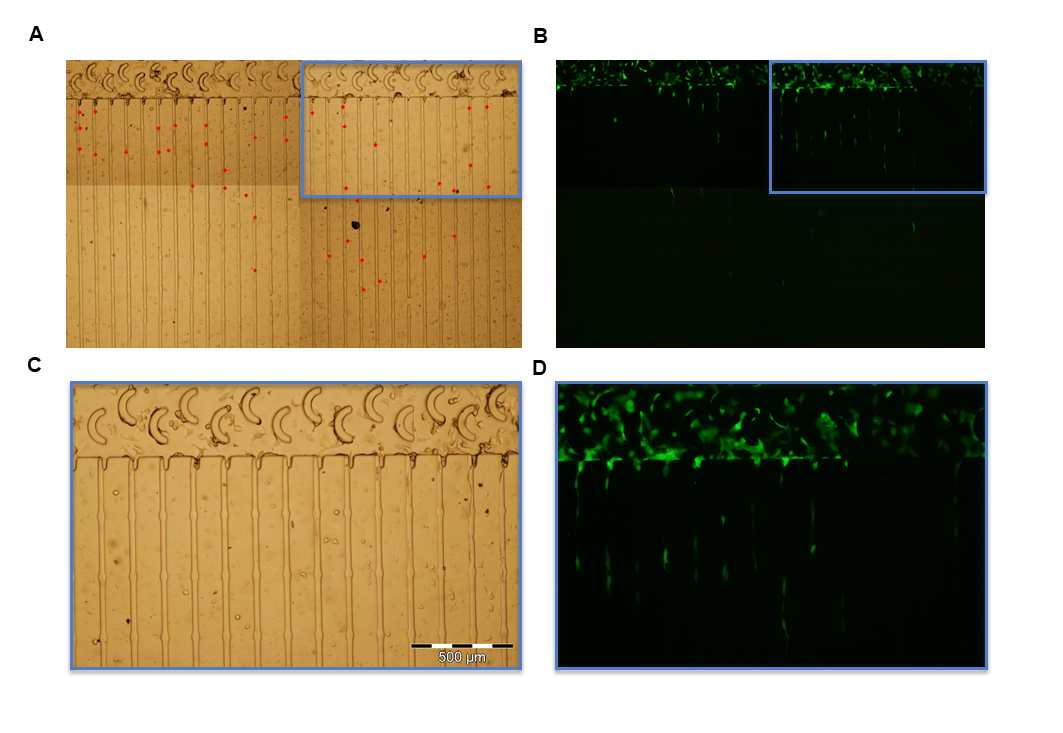
Figure S6.** The cell migration process in the microfluidic chip, the green color shows the live cells. A) The bright-field picture of the microfluidic chip in which the cells were shown by red spot and C) a close view of the microfluidic chip. (B) The fluorescence picture of the microfluidic chip in which the cells were shown by green color.


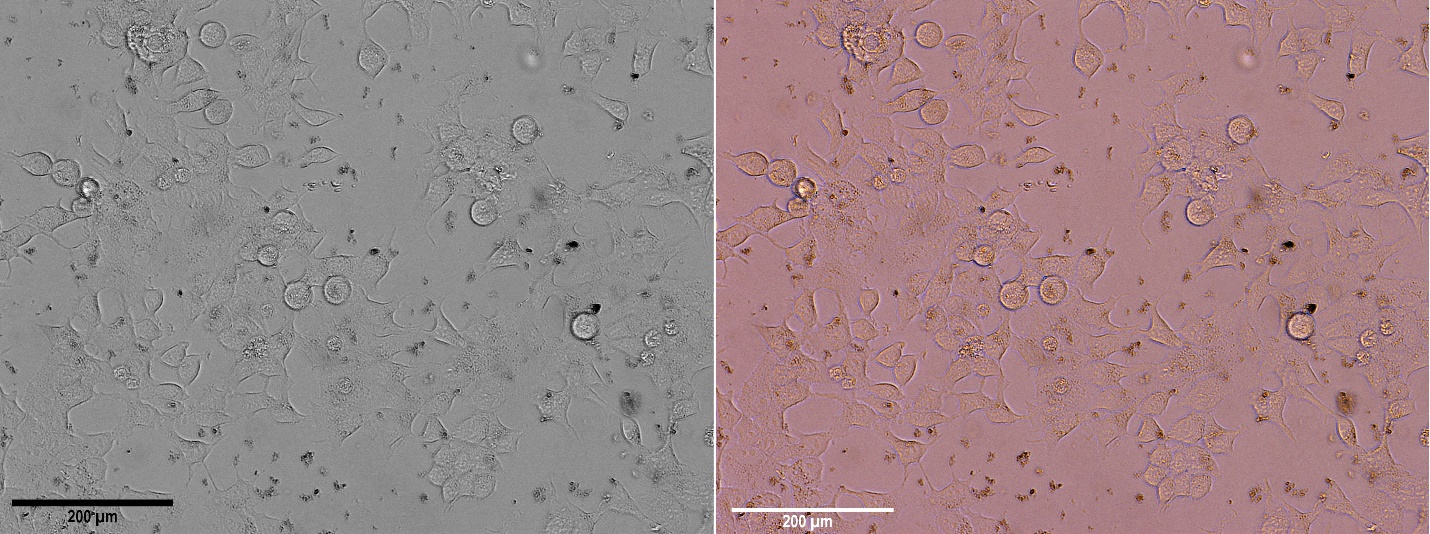


**Figure S7.** The Bright field pictures of MCF-7 cells treated by GO (10 µg.mL^-1^ ) showed the distribution of GO nanosheets on cells.


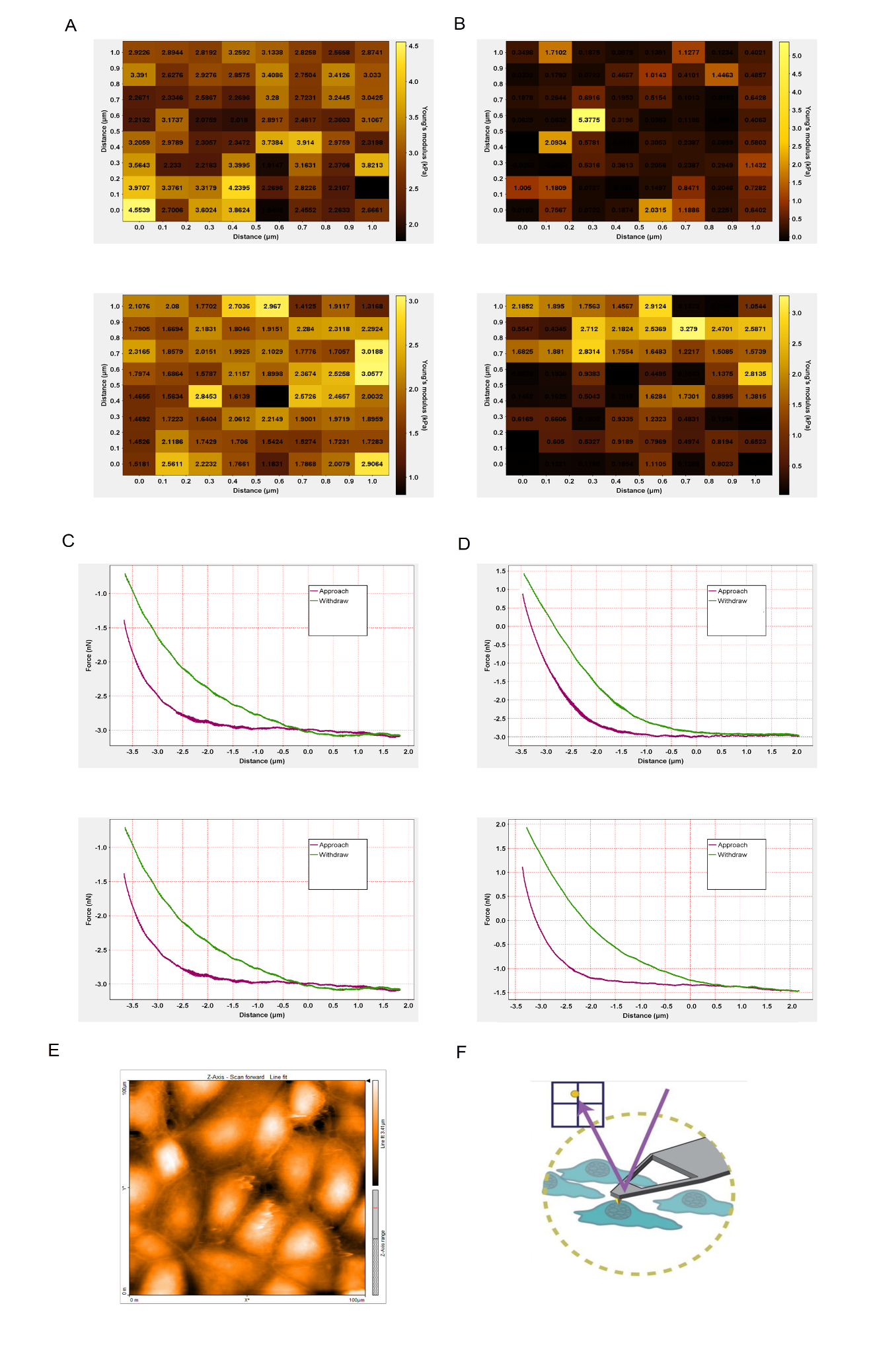


**Figure S8.** A) The graphic data of Young’s modulus of a region of the cell from the MCF7 cell line, B) The graphic data of Young’s modulus of a region of the cell from the MDA-MB-231 cell line, C) The Force-Distance curve of the cells from the MCF7 cell line, D) The Force-Distance curve of the cells from the MDA-MB-231 cell line, E) The picture of the cells from MCF7 cell line F) The schematic illustration of The Young’s module measurement by AFM.

1. Mehrdad-Vahdati, B., et al., *A novel aspect of functionalized graphene quantum dots in cytotoxicity studies.* Toxicology in Vitro, 2019. **61**: p. 104649.
